# Prevalence and genotype screening of human papillomavirus among women attending a private hospital in Northern Cyprus: an 11-year retrospective study

**DOI:** 10.1186/s12905-023-02451-8

**Published:** 2023-06-03

**Authors:** Buket Baddal, Makbule Naz Oktay, Aysegul Bostanci, Mufit Cemal Yenen

**Affiliations:** 1 Department of Medical Microbiology and Clinical Microbiology, Faculty of Medicine, Near East University, Nicosia, 99138 Cyprus; 2 Department of Molecular Biology and Genetics, Faculty of Arts and Sciences, Eastern Mediterranean University, Famagusta, Cyprus; 3 Molecular Microbiology Laboratory, Near East University Hospital, Nicosia, 99138 Cyprus; 4 Department of Gynecology and Obstetrics, University of Kyrenia Hospital, Kyrenia, Cyprus

**Keywords:** Human papillomavirus, High-risk HPV, Cervical cancer, Genotyping, Real-time PCR, Cyprus

## Abstract

**Background:**

Human papillomavirus (HPV) is the most common sexually transmitted pathogen both in men and women. Accumulating epidemiological evidence supports a strong association between HPV infection and cancer of the cervix, vulva, vagina, anus, and penis. Currently, data on the HPV prevalence and genotyping is lacking in Northern Cyprus, a region in which HPV vaccination is not freely accessible via the national immunization program. The aim of this study was to evaluate the HPV type-specific prevalence in women with and without cytological abnormalities living in Northern Cyprus.

**Methods:**

A total of 885 women who presented to the Department of Gynecology and Obstetrics Clinic between January 2011 and December 2022 were included in the study. Samples were collected for cytology. Cervical specimens were investigated for the presence of HPV-DNA and genotyping of HPV was performed using real-time polymerase chain reaction (rtPCR). Cytological examination was interpreted according to the Bethesda system.

**Results:**

Among all patients, overall high-risk HPV DNA prevalence was 44.3%. HPV-16 and HPV-18 positivity was found in 10.4% and 3.7% of women respectively, while other high-risk HPV (OHR-HPV) was the most frequent type of HPV (30.2%). The highest frequency of HPV infection was observed in the 30–55 age group (51.0%), followed by the < 30 age group (45.7%). Co-infection with two or more HPV types was observed in 17.0% of all positive samples, in which the prevalence of HPV-16 + HPV-18 was 2.3%, HPV-16 + OHR-HPV and HPV-18 + OHR-HPV was 12.0% and 5.1%, respectively. Among the screened patients, 37.5% had abnormal and 62.5% had normal cytology results. HR-HPV positivity was 65.7% and 34.0% in patients with abnormal and normal cytology. The highest incidence of HRC-HPV was OHR-HPV types (44.7%) in positive cytology cases. Among women with a cytology result of ASCUS, L-SIL, H-SIL and unspecified dysplasia, 52.1%, 67.6%, 97.5% and 75.6% were respectively infected with HR-HPV.

**Conclusion:**

The present study provides the latest epidemiological data related to HPV prevalence and genotype distribution among women living in Northern Cyprus. Considering the unavailability of free vaccination in the community, it is imperative to implement local HPV screening programs and provide guidelines on HPV prevention and measures during early school education.

## Introduction

Cervical cancer was ranked as the fourth most prevalent cancer type and the fourth leading cause of cancer related deaths in women with an estimated 604,000 new cases and 342,000 deaths being reported on GLOBOCAN2020 [1]. Human papillomavirus (HPV) is a virus that is mainly sexually transmitted via skin-to-mucosa or skin-to-skin contact. A number of studies have documented non-sexual routes of transmission such as from mother to child via contact with genital mucosa during natural birth, or possible transmission from contaminated surfaces or medical equipment [2]. HPV is a known agent in the development of cervical cancer among other anogenital cancers including penis, anus, vulva, and vagina [3]. Currently, over 200 hundred different types of HPV have been identified which are classified as low risk (LR-HPV) or high-risk (HR-HPV) based on their oncological potential. HPV-16, 18, 31, 33, 35, 39, 45, 51, 52, 56, 58, 59, 68 have been identified as high risk by the Agency on Research Cancer. More than 96% of cervical cancer cases have been linked with a persistent infection of one of the thirteen HR-HPV types, with HPV-16 and HPV-18 being responsible for 71% of cases [4, 5]. On the other hand, while most LR-HPV infections are often asymptomatic and cleared within a year by the host immune system, some may present with clinical symptoms such as genital warts and laryngeal papillomas (HPV 6–11) [6].

Transmission risk factors of the virus are primarily associated with sexual behaviors such as engaging in unprotected sex, having multiple partners, having non-monogamous male partners and starting sexual activity at an early age. Nonetheless, not receiving vaccination prior to engaging in sexual activity, a weakened immune system and exposure to other sexually transmitted infections also escalate the likelihood of contracting the infection and developing cervical lesions. Apart from these, long-term use of oral contraceptives, belonging to Black or Hispanic ethnic groups and having a history of chlamydia infection have also been linked to a higher risk of contracting HPV [7, 8]. In terms of HPV recurrence, diagnosis of cervical intra-epithelial neoplasia 3 (CIN3) instead of CIN2 as well as a positive endocervical margin are among the factors associated with increased risk of persistence/recurrence [9].

Considering that HPV infection leads to the development of cervical cancer, routine HPV screening and genotyping can be implemented as a preventative measure against cervical cancer as well as other HPV-related cancers. Routine screening can also reduce the incidence and mortality rate of cervical cancer by early diagnosis and treatment of cervical pre-cancers. A study performed on the trends of cervical cancer incidence and mortality suggested that the decrease in the incidence and mortality rates were associated with the improvement of cervical cancer screenings [10]. In Northern Cyprus, there is no guideline and regular screening program for HPV. The Ministry of Health in Northern Cyprus started public cervical cancer screening in 2022 and performs HPV screening for participants. In the Republic of Cyprus, there are no established screening protocols for cervical cancer, and the screening is carried out based on recommendations by doctors [11].

Data obtained from HPV genotyping schemes can aid in nationwide vaccination programs. As of February 2023, there are three HPV vaccines that have been approved by the United States Food and Drug Administration (FDA). The first commercially available HPV vaccine was the quadrivalent HPV vaccine that targets four HPV types, HR-HPV-16 and HPV-18 together with LR-HPV-6 and HPV-11, which are responsible for 90% of genital warts. Bivalent HPV vaccine was first approved by European Medicines Agency (EMA) in 2007 and was later approved by FDA in 2009. Cervarix only offers protection for HPV-16 and HPV-18. The most recent HPV vaccine, nine-valent Gardasil 9 was approved by the FDA in 2014, and offers protection against HPV 6, 11, 16, 18, 31, 33, 45, 53 and 58. The additional five genotypes could mean that Gardasil 9 may potentially be effective against 90% of cervical cancers [12]. In January 2016, Republic of Cyprus added HPV vaccines to the national immunization program, while in Turkish Republic of Northern Cyprus (TRNC) HPV vaccination is still not included within the national immunization scheme and is only received on a personal basis with a cost. Albeit, the HPV vaccination is included in the Ministry of Health’s adult vaccination guide. While it is recommended for women to get vaccinated between the ages of 19–26, for men it is recommended to get vaccinated between the ages of 19–21, and between the ages of 22–26 for male individuals who have risk factors or different indications [13]. Before the pandemic, the free vaccination program was initiated by the Ministry of Health for 12-year-old girls, but it has been disrupted due to COVID-19. It has been reported that the vaccination programs in schools have resumed in selected pilot schools after the pandemic.

In this study our aim was to provide insights into the prevalence of HPV in Northern Cyprus, a region in which free immunization is not accessible, by documenting the HPV frequency and distribution of HPV-16, HPV-18 and other HR-HPV among women attending a private hospital between the years 2011 and 2022.

## Materials and methods

### Ethical approval

This study was performed following the Declaration of Helsinki and was approved by the Institutional Review Board at Near East University Hospital (YDU/2022/106–1607). The study was exempt from patient informed consent as all data used was generated during diagnostic HPV genotyping tests of cervical swab samples which were referred to the Molecular Microbiology Laboratory at NEU Hospital by experienced gynecologists as part routine check-up.

### Study population

Women aged 16 years and older who had been admitted to gynecological clinics at Near East University Hospital, Cyprus for routine cervical cancer screening between January 2011 and December 2022 were included in the retrospective study. Women with a known diagnosis of cervical cancer, immunosuppression or referral for an abnormal cervical sample were excluded.

The study included cervical swab specimens collected by experienced clinicians using FLOQSwabs in eNAT medium (Copan, Bescia, Italy) for HPV DNA detection. Each participant had a gynecologic examination including liquid-based cytology and a cervical sample for HPV detection. ThinPrep Pap test specimens were collected by inserting a cytobrush into the endocervical canal, which was which was immediately placed in methanol containing vials of Thin Prep transport medium. The specimens were stored at 15 to 20 °C and transported to the laboratory within 24 h of collection. A ThinPrep 2000 processor (Cytyc Corporation, USA) was used to prepare slides which were stained with Papanicolaou stain. Cervical cytological samples were classified based on the Bethesda system classification: atypical squamous cells of undetermined significance (ASCUS), low-grade squamous intraepithelial lesion (L-SIL) or high-grade squamous intraepithelial lesion (H-SIL). Cytology results as well as demographic, clinical and laboratory data of patients were collected and investigated.

### DNA extraction and HPV detection by rtPCR

HPV DNA was extracted from the cervical swabs using GeneAll Ribospin vRD DNA/RNA Extraction Kit (GeneAll Biotechnology, Korea), according to manufacturer’s instructions. Glass fiber membrane spin columns were used to purify nucleic acids for rtPCR analysis. The isolated DNA was immediately processed for the detection and typing of HPV DNA.

HPV DNA amplification was performed using the commercial HPV Genotypes 14 Real-TM Quant Real-Time PCR Kit (Sacace Biotechnologies, Italy) until 2018 and QIAscreen HPV PCR Detection Kit (Qiagen, Germany) after 2018 on the Rotor-Gene® Q instrument (Qiagen, Germany). The HPV Genotypes 14 Real-TM Quant Real-Time PCR Kit can detect HPV 16, 18, 31, 33, 35, 39, 45, 51, 52, 56, 58, 59, 66 and 68, and targets viral genome region E6 and E7. The QIAscreen HPV PCR Detection Kit is directed against the E7 gene of the HPV genome. The PCR test can detect 15 high-risk HPV (HR-HPV) types in a single analysis by providing individual results on the HR genotypes, HPV-16 and HPV-18, and pooled results on the other HR genotypes (OHR-HPV) including 31, 33, 35, 39, 45, 51, 52, 56, 58, 59, 66, 67, and 68. Briefly, viral nucleic acid products obtained were amplified by distributing 15 µl of QIAscreen master mix and 5 µl for each extracted sample DNA into tube strips. A sample control (human β-globin) and a negative control were used to monitor experimental process and minimize sampling errors. Results of rtPCR were interpreted as following: HPV positive: Ct values are < 36 for HPV-16 and/or HPV-18 and/or < 33.5 for OHR-HPV types (regardless of β-globin Ct value); HPV negative: Ct value is ≤ 30 for β-globin and Ct values are ≥ 36 for HPV-16 and HPV-18 or when there is no signal and when Ct value is ≥ 33.5 for the OHR-HPV types or when there is no signal. For the Sacace HPV Genotypes 14 Real-TM Quant Real-Time PCR Kit, PCR amplification was performed using 15 µl of each mastermix (total of 4 mixes) and 10 µl of the extracted DNA per mix. Results were interpreted as following: HPV positive: Ct values are < 33 for all HPV types, and Ct value for internal control (human β-globin) is < 30. Only qualitative results were taken into consideration.

### Statistical analysis

Statistical analysis was performed using the Statistical Package for the Social Sciences (SPSS) version 22.0 software (SPSS Inc., Chicago, IL, USA). HPV prevalence was determined in all samples and the type-specific distribution of HPV-positive samples was analyzed in the context of different age-groups. The association between HPV infection and specific genotypes with different cytology results (ASCUS, L-SIL, H-SIL) was assessed for all samples for which the information was available.

Pearson chi-square (χ^2^) test was used to compare categorical measurements between different groups. The distribution of normal variables was analyzed using the Student’s *t-*test. Values of *p* < 0.05 were considered statistically significant.

## Results

### HPV prevalence and age distribution

**Fig. 1:**
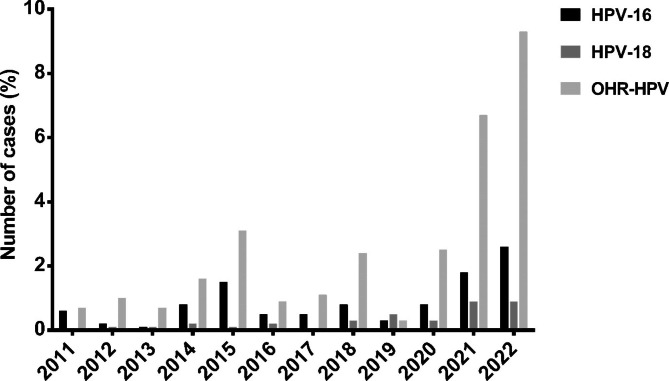
Annual prevalence of HPV-16, HPV-18 and OHR-HPV between 2011–2022

A total of 885 female patients were included in this study. Among all patients, 392 of them (44.3%) were high-risk HPV DNA positive. HPV-16 and HPV-18 positivity was detected in 92 (10.4%) and 33 (3.7%) of the patients respectively, while other high-risk HPV (OHR-HPV) was the most frequent type of HPV being positive in 267 (30.2%) of the patients screened. In 493 (55.7%) of the patients, no evidence of HPV-DNA was found. The annual distribution of HPV-16, HPV-18 and OHR-HPV are given in Fig. 1 which show an increasing trend of OHR-HPV.

Co-infection with two or more HPV types was observed in 67 (17.0%) of all positive samples, in which the prevalence of HPV-16 + HPV-18 was 9 (2.3%), HPV-16 + OHR-HPV and HPV-18 + OHR-HPV was 47 (12.0%) and 20 (5.1%), respectively.

**Fig. 2:**
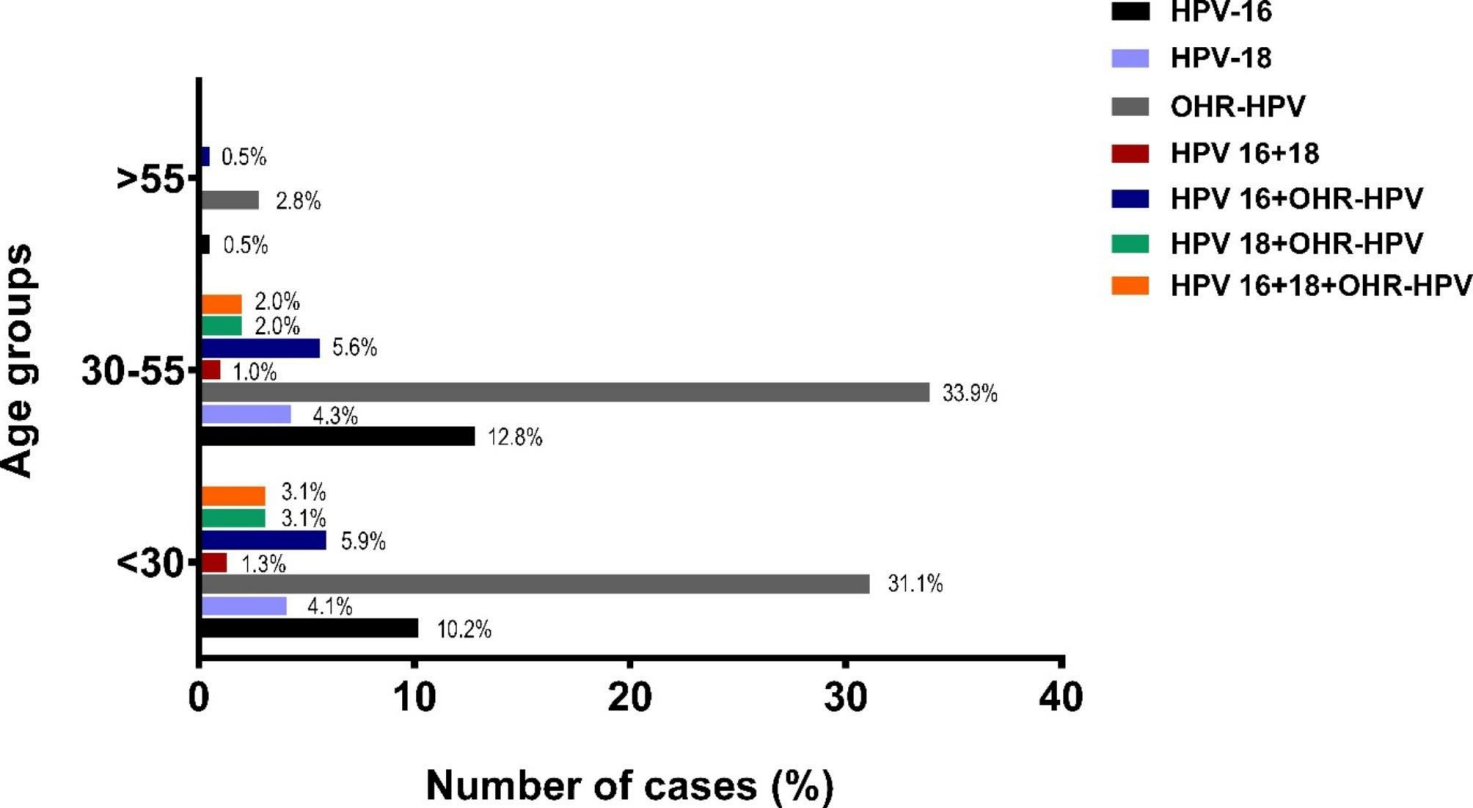
Distribution of HPV-16, HPV-18 and OHR-HPV types according to the age of tested persons, during 2011–2022

The mean age of patients was 33.8 ± 9.72 (min. 16, max. 81). The patients were evaluated in three age groups; <30, 30–55, and > 55. The highest frequency of HPV infection was observed in the 30–55 age group (n = 200, 51.0%), followed by the < 30 age group (n = 179, 45.7%) as shown in Fig. 2. The lowest frequency of HPV positivity was reported in the > 55 age group (n = 13, 3.3%). The distribution of HPV positivity in different age groups was statistically significant in OHR-HPV positive patients (*p* = 0.009) (HPV-16, *p* = 0.642, HPV-18, *p* = 0.370).

### HPV genotypes and cervical cytology

Among the patients included in the study, 37.5% (n = 332) had abnormal and 62.5% (n = 553) had normal cytology results. HR-HPV positivity was 65.7% (n = 218) and 34.0% (n = 188) in patients with abnormal and normal cytology, respectively. The highest incidence of HRC-HPV was other high-risk (OHR) HPV types (44.7%) in positive cytology cases, followed by HPV-16 (17.5%) and HPV-18 (4.8%). The distribution of HPV in women with normal cytology was HPV-16 7.1%, HPV-18 3.6% and OHR-HPV 23.3%.

The highest number of patients with cervical lesions belonged to ASCUS (15.8%, n = 140) and L-SIL (18.2%, n = 161), followed by H-SIL (4.5%, n = 40) and unspecified dysplasia (4.6%, n = 41). Among women with a cytology result of ASCUS, L-SIL, H-SIL and unspecified dysplasia, 52.1%, 46.6%, 97.5% and 75.6% were infected with HR-HPV, respectively (Table 1).

**Table 1:** Distribution of HPV-16, HPV-18 and OHR-HPV types according to the severity of cervical lesions in women with positive cytology, during 2011–2022

HPV Genotype	ASCUS *n*, (%)	LSIL *n*, (%)	HSIL *n*, (%)	Unspecified dysplasia* *n*, (%)	Total
HPV-16	19 (5.7)	15 (4.5)	14 (4.2)	10 (3.0)	58
HPV-18	4 (1.2)	8 (2.4)	2 (0.6)	2 (0.6)	16
OHR-HPV	50 (15.1)	52 (15.7)	23 (7.0)	19 (5.7)	144
Negative	67 (20.2)	86 (25.9)	1 (0.3)	10 (3.0)	164
Total	140	161	40	41	382

Abbreviations: ASCUS, atypical squamous cells of undetermined significance; LSIL, low-grade squamous intraepithelial lesion; HSIL, high-grade squamous intraepithelial lesion; OHR-HPV, other high‐risk genotypes

* Dysplasia of cervix uteri, unspecified

128 patients with normal cytology results were reported to have no symptoms and visited the clinic for a control check-up, while 192 of the HR-positive patients attended the gynecology clinic for other symptoms including inflammation, discharge, rash, vaginitis, cervicitis, vulvitis, condyloma, leukoplakia, cervical ectropion, uterine polyps, postcoital bleeding and pelvic pain.

## Discussion

The present study is an 11-year retrospective study on the HPV prevalence and distribution among women attending a private hospital in Northern Cyprus between 2011 and 2022. Among the total of 855 patients, overall HPV prevalence was 44% (n = 392). HPV 31, 33, 35, 39, 45, 51, 52, 56, 58, 59, 66, 68 were all categorized as OHR-HPV and were the most common types being detected in 30.2% of the screened patients followed by HPV-16 (10.4%) and HPV-18 (3.7%). The annual prevalence of OHR-HPV was observed to have an increasing trend.

The results of the current study (44%) show a lower prevalence than a previous study performed in Cypriot women in 2017 which was reported to be 72.8% [14]. The difference between the results could be related to the fact that the patients involved in the 2017 study consisted of women already experiencing some degree of cervical cytological abnormalities associated with HPV infection. A recent study by Zhang et al. reported a considerable difference between HPV prevalence of in-clinic and general healthy population [15]. The target population in the current study consisted of both patients with abnormal cytology (37.5%) and women from the general population with normal cytology (34.0%) who attended the hospital for regular check-ups or other unrelated symptoms, in which HPV positivity was detected to be 65.7% and 34.0%, respectively. The results of our study are consistent with recent studies performed in different parts of the world that reported HPV prevalence between 14.0% and 67.6% [16–20]. The wide range can be explained by factors including the availability of immunization programs, socioeconomic status of the country, education levels and other risk factors.

HPV-16 and HPV-18 are the most carcinogenic HPV types, responsible for 70% of all cervical cancer cases [4, 5]. Similar to the results from the current study (HPV-16: 10.4%, HPV-18: 3.7%), a previous study performed in the Republic of Cyprus reported HPV-16 and HPV-18 prevalence of 17.7% and 4.1%, respectively [14]. HPV-52 and HPV-58 that are included in the OHR-HPV group were among the most common types of HPV observed according to recent literature [15–17, 21].

Co-infection with two or more HPV types were detected in 17.0% of the positive patients with the most common co-infection being HPV-16 + OHR (12.0%) followed by HPV-18 + OHR (5.1%) and HPV-16 + HPV-18 (2.3%). Co-infection with more than one HPV type is a common phenomenon which is heavily influenced by life-style differences such as smoking and multiple sexual partners [22]. The co-infection rate obtained in the current study is slightly lower than the average of ~ 27.46% which has been reported by several recent studies [14, 15, 23].

Among the 885 women, HR-HPV prevalence was found to be highest in age groups between 30 and 55 with 51.50% of all positive samples consisting of this age group followed by women under the age of 30 (45.7%). In a study performed among 4267 Turkish women, Altay-Kocak et al. also reported the 35–55 age group to have the highest frequency of HR-HPV [16]. In contrast to our results, the study undertaken in 596 women in Republic of Cyprus indicated HR-HPV prevalence peaking at younger ages in which 84.7% of HR-HPV positivity have been detected in individuals below the age of 25 [14]. Type specific prevalence rates remain constant throughout age groups < 30 and 30–55 among studies in literature, with the majority of positive results belonging to the OHR-HPV group followed by HPV-16 and HPV-18.

Among patients with cytological abnormalities, ASCUS (15.8%, n = 140) and L-SIL (18.2%, n = 161) were the most common cervical lesions followed by unspecified dysplasia (4.6%, n = 41) and H-SIL (4.5% n = 40). HR-HPV prevalence among individuals with cervical abnormalities were 52.1%, 46.6%, 75.6% and 97.5%, respectively, showing a correlated increase between HR-HPV infection and severity of cytological abnormalities. This trend was also described in the Republic of Cyprus (ASCUS: 74%, L-SIL: 85.9%, H-SIL: 82.6) [14] and in China (ASCUS: 30.8%, L-SIL: 36.5%, H-SIL 54.9%) [24]. HR-HPV prevalence was particularly high in patients with H-SIL in accordance with 84% HPV prevalence in H-SIL cases in Europe and 85% globally reported in a meta-analysis by Guan et al. [25].

Prevention strategies of cervical cancer can be divided into two parts. Primary prevention focuses on avoidance of HPV infection and vaccination. Main risk factors of HPV infection include multiple sexual partners, unprotected sexual intercourse and polygamy therefore abstinence, mutual monogamy or using protection such as condoms can be effective against HPV infections [26]. A recent study has suggested the use of a prognostic nomogram for the prediction of persistence and recurrence of cervical dysplasia after primary colonization [27]. HPV vaccines have also proven to be very effective in the prevention of HPV infections and HPV-related cancer progression Currently, three highly effective vaccines against HPV are available; Gardasil, Cervarix, Gardasil 9. A substantial reduction in H-SIL and cervical cancer rates were reported following the introduction of routine HPV vaccination in England [28]. HPV vaccines have been included in the national immunization program in the Republic of Cyprus since January 2016. Secondary prevention includes routine screenings for cervical cancer as well as HPV. Routine HPV screening is strongly recommended by the World Health Organization as a preventative measure for the development of cervical pre-cancers such as ASCUS, L-SIL and H-SIL and cervical cancer [29]. In a 2023 study performed among adults between 18 and 45 years of age living in Northern Cyprus, the authors reported that the participants were not aware of the transmission routes of HPV, and did not have accurate knowledge regarding early diagnosis and screening or HPV vaccination [30]. Considering this, health policies should be developed by national health departments to increase the awareness of individuals about HPV, the importance of routine screening as well as to provide education and access to free vaccination.

Treatment options for cervical cancer depends on the stage of the disease. There are two treatment options available for pre-cancerous growth which focus on killing the abnormal cell tissue before it can progress into invasive cancer. These procedures include conization where a cone shaped cut is made to remove the abnormal cell tissue for diagnosis or for treatment in some cases where all of the abnormal tissue is removed. In more severe cases where the cancerous growth has evolved into invasive cancer, hysterectomy is required. For early stages of cervical cancer, simple hysterectomy is performed in which only the uterus and cervix is removed, and vagina and parametria and uterosacral ligaments are left intact. More severe cases require radical hysterectomy where a small part of upper vagina, parametria uterosacral ligaments are removed as well as the uterus and cervix. Both types of hysterectomy results in infertility and may also cause varying levels of dysfunction in urinary and intestinal systems [31]. Different approaches for hysterectomy include abdominal hysterectomy where the uterus and cervix are removed from a large abdominal incision, and laparoscopic hysterectomy where small instruments and a camera is inserted into the abdomen through small incisions. Although the latter choice is less invasive and may in turn cause less complications, recent research shows a higher recurrence rate for laparoscopic hysterectomy [32]. Radical trachelectomy is a primary method of treatment for patients with early-stage cervical cancer [33] and is considered an option where the patient’s fertility is protected by removing the cervix, small part of upper vagina and parametria but leaving the uterus intact. This procedure can be performed via abdomen, vagina or using laparoscopy [34].

The current study has important strengths. It targets women in an understudied area in Northern Cyprus from which limited data on HPV is available. The study also covers an 11-year period and therefore makes a significant contribution to the literature. The study also has certain limitations. One limitation of the study was that individual detection of OHR-HPV types was not possible, therefore the dominant HPV types could not be stated. Another limitation could be the sample population which mostly consisted of patients that had attended the hospital as a consequence of having HPV related symptoms or cervical abnormalities, which could lead to selection bias. The vaccination status and education level of the participants were unknown and the number of patients above the age of 55 was considerably less compared to other age groups. Future studies on HPV prevalence in a population with known vaccination status and comprehensive genotyping will provide new insights towards the epidemiology and prevention of HPV in the country.

## Conclusions

This study reports data on the HPV prevalence and genotype distribution in Northern Cyprus population that could provide valuable data which can be used in developing further prevention strategies while highlighting the importance of vaccination and early diagnosis of cervical pre-cancers with HPV DNA testing.

## Data Availability

The data that support the findings of this study are available upon request from the corresponding author.

## Abbreviations

ASCUS: Atypical squamous cells of undetermined significance
FDA: Food and Drug Administration
HPV: Human papillomavirus
HR-HPV: High risk-human papillomavirus
H-SIL: High-grade squamous intraepithelial lesion
LR-HPV: Low risk-human papillomavirus
L-SIL: Low-grade squamous intraepithelial lesion
rtPCR: Real-time polymerase chain reaction
TRNC: Turkish Republic of Northern Cyprus
